# Nanobody engineering: computational modelling and design for biomedical and therapeutic applications

**DOI:** 10.1002/2211-5463.13850

**Published:** 2024-06-19

**Authors:** Nehad S. El Salamouni, Jordan H. Cater, Lisanne M. Spenkelink, Haibo Yu

**Affiliations:** ^1^ Molecular Horizons and School of Chemistry and Molecular Bioscience University of Wollongong Australia; ^2^ ARC Centre of Excellence in Quantum Biotechnology University of Wollongong Australia

**Keywords:** artificial intelligence, machine learning, molecular dynamics simulations, nanobody, quenchbody, structure prediction

## Abstract

Nanobodies, the smallest functional antibody fragment derived from camelid heavy‐chain‐only antibodies, have emerged as powerful tools for diverse biomedical applications. In this comprehensive review, we discuss the structural characteristics, functional properties, and computational approaches driving the design and optimisation of synthetic nanobodies. We explore their unique antigen‐binding domains, highlighting the critical role of complementarity‐determining regions in target recognition and specificity. This review further underscores the advantages of nanobodies over conventional antibodies from a biosynthesis perspective, including their small size, stability, and solubility, which make them ideal candidates for economical antigen capture in diagnostics, therapeutics, and biosensing. We discuss the recent advancements in computational methods for nanobody modelling, epitope prediction, and affinity maturation, shedding light on their intricate antigen‐binding mechanisms and conformational dynamics. Finally, we examine a direct example of how computational design strategies were implemented for improving a nanobody‐based immunosensor, known as a Quenchbody. Through combining experimental findings and computational insights, this review elucidates the transformative impact of nanobodies in biotechnology and biomedical research, offering a roadmap for future advancements and applications in healthcare and diagnostics.

## Graphical abstract

This review focuses on structural characteristics, functional properties, and computational approaches driving the design and optimisation of synthetic nanobodies. We discuss recent advancements in computational methods for nanobody modelling, epitope prediction, and affinity maturation, shedding light on their intricate antigen‐binding mechanisms and conformational dynamics. It underscores their advantages over conventional antibodies from a biosynthesis perspective in diagnostics, therapeutics, and biosensing.

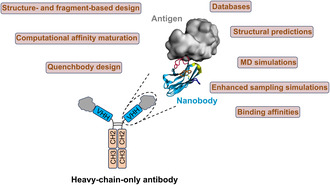

**Abbreviations**
CDR: complementarity‐determining region
CH: constant heavy constant domains
CL: constant light constant domains
CQ‐body: coiled quenchbody
DSMBind: denoising score matching for binding energy prediction
FAB: fragment‐of‐antigen‐binding
GaMD: Gaussian accelerated molecular dynamics
GPCR: G‐protein‐coupled receptor
INDI: integrated database of nanobodies for immunoinformatics
MD: molecular dynamics
MM/GBSA: molecular mechanics/generalised born surface area
MM/PBSA: molecular mechanics/Poisson Boltzmann surface area
MW: molecular weight
NanoBRET: NanoLuciferase‐based bioluminescence resonance energy transfer
PC: principal component
PD: programmed death 1
PD‐L1: programmed death ligand 1
Q: fraction of native atomic contacts
Q‐body: quenchbody
RMSD: root‐mean‐squared displacement
SAbDab: structural antibody database
SARS‐CoV‐2: severe acute respiratory syndrome coronavirus 2
scFv: Single‐chain variable fragment
*T*_m_
: melting temperature
VH: heavy‐chain variable domain
VHH: variable domain of the heavy chain of heavy‐chain‐only antibodies
VIMAS: Venn‐intersection of multi‐algorithms screening
VL: light‐chain variable domain

Conventional antibodies, found in humans and other mammals, are Y‐shaped molecules consisting of multiple domains (Fig. 1). Each antibody consists of two identical heavy chains, comprising VH, CH1, CH2 and CH3 domains and two identical light chains comprising VL and CL domains. The heavy‐chain constant domains CH2 and CH3 constitute a crystallisable fragment, known as Fc. The arms of the Y‐shaped structures are called Fragment‐of‐antigen‐binding (Fab) regions and consist of two heavy‐chain variable domains (VH) and two light‐chain variable domains (VL) along with two constant regions (CH1 and CL). The antigen‐binding site, also referred to as the paratope, is situated at the tips of the antibody arms, within a pocket or groove created by the variable domains of the heavy (VH) and light chains (VL). These binding sites consist of six hypervariable loops characterised by differing lengths and amino acid compositions, commonly referred to as complementarity‐determining region (CDR) loops. Recombinant antibody fragments, such as Fab and single‐chain variable fragments (scFv), as well as nanobodies (Fig. 1) and polyvalent engineered variants [1], are gaining recognition as effective antigen binders [2]. These fragments maintain the targeting specificity of complete monoclonal antibodies while offering distinctive and superior properties suitable for various diagnostic and therapeutic applications [2, 3, 4]. They are used as tracers for diagnostic *in vitro* imaging [3] and diagnosis [5], biosensors [6] as well as developing therapeutics targeting various diseases including cancers [2, 7, 8] and SARS‐CoV‐2 [9].

**Fig. 1:**
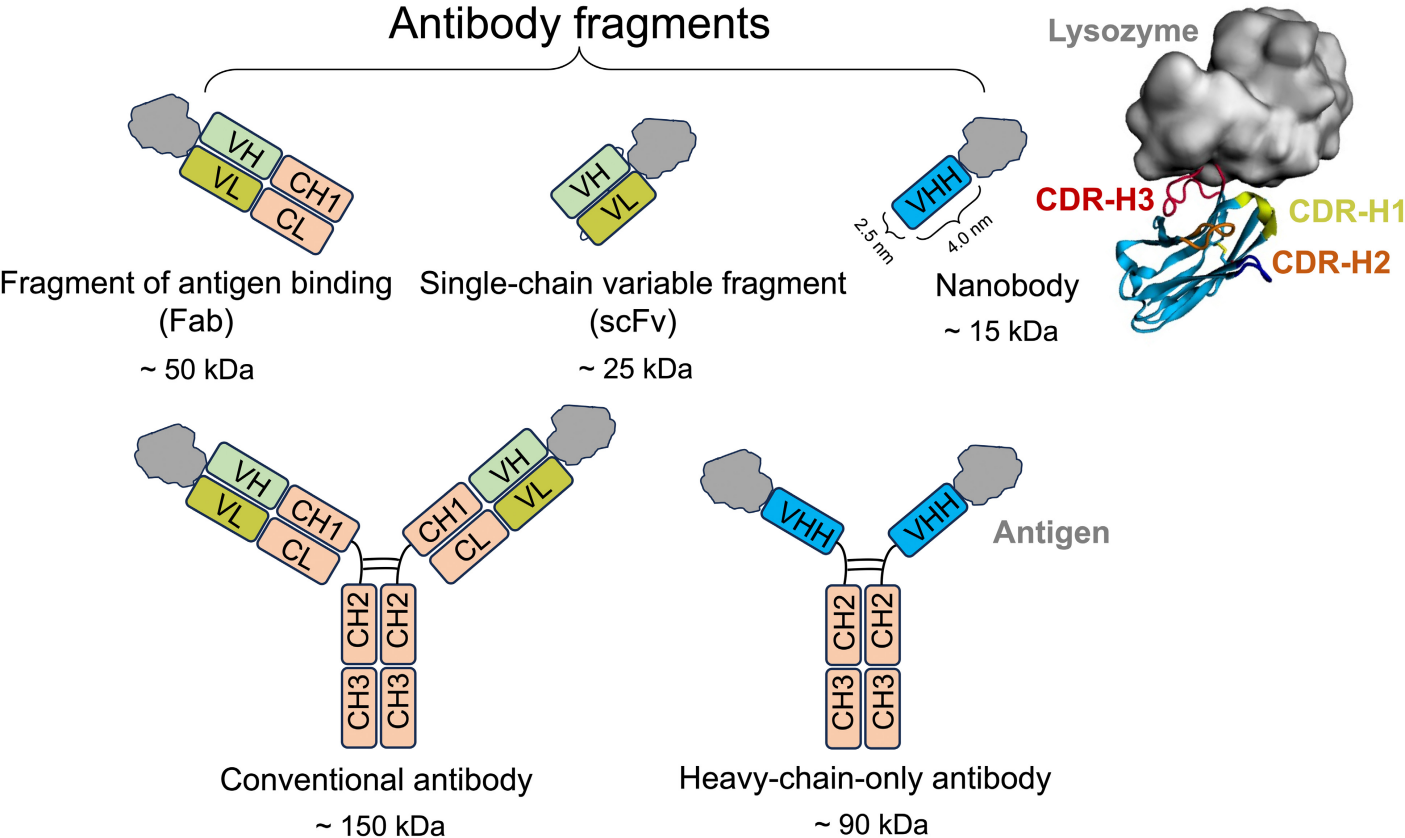
Graphical representations of a conventional antibody, a heavy‐chain‐only antibody and a nanobody as well as antibody fragments. A three‐dimensional structure of a nanobody (cyan) binding lysozyme (grey), illustrating the three CDR loops and the disulphide bond (PDB ID: 1ZVH).

Nanobodies, also referred to as VHH or single‐domain antibodies due to lacking light chains, are small antibody fragments derived from heavy‐chain‐only antibodies. They were first reported in 1993 in camel serum [10], but they are also found in other camelids such as llamas and alpacas, as well as sharks [11]. They only have three CDR loops and their variable single domains alone are sufficient for binding to antigens, with binding affinities comparable to those of monoclonal antibodies [10]. Nanobodies are even capable of interacting with hidden epitopes buried in clefts on protein surfaces that are inaccessible to conventional VH‐VL pairs [12], such as those found in enzyme active sites [13] and in the SARS‐CoV‐2 spike protein [14]. Like antibodies, nanobodies contain immunoglobulin‐like β‐sandwich scaffolds that consist of antiparallel β‐strands arranged into two sheets and held together by an internal disulphide bond (Fig. 1). This structural framework provides stability for the three CDRs that make up the binding surface which can be considered synonymous with the paratope of conventional antibodies [15]. These CDR loops have variable lengths, composition, and structure, with CDR‐H3 being the most variable in sequence, with lengths ranging from 12 to 18 residues [16], and is therefore considered one of the largest contributors to antigen‐binding specificity [12, 17]. Notably, nanobodies typically possess longer CDR‐H3 loops than antibodies and can adopt a linked or extended conformations, allowing nanobodies to access binding pockets with finger‐like antigen‐binding paratopes not accessible to the short loop counterparts found in the heavy‐ and light‐chain paratopes of conventional antibodies [16]. As a result of this, CDR‐H3 loops in nanobodies occupy a larger conformational space than those in antibodies as they are not restricted by the paired light‐chain domain [18]. The CDR‐H3 of a nanobody typically contributes to over 50% of all binding interactions within the entire paratope [19, 20]. The rest of the nanobody is composed of four regions whose sequences and structures are more conserved than those in conventional antibodies and are referred to as the framework regions [21]. The hydrophilic nature of the region in nanobodies that corresponds to the VH‐VL interface in conventional antibodies exceeds that observed in other antibody fragments like Fab and scFv. This characteristic mitigates self‐association or dimerisation, thereby ensuring the preservation of nanobodies in a monomeric state [11, 22]. In general, nanobody paratopes exhibit greater diversity in the structural segments, the residues used for antigen interaction, and the variety of contacts established with the antigen, than the paratopes of conventional antibodies [20].

Nanobodies offer several advantages compared to conventional antibodies (MW: ~ 150 kDa), including their small size of ~ 110 amino acids (MW: ~ 12–15 kDa), stability, and solubility, while retaining the ability to bind to their targets with high affinities, similar to antibodies [11]. Additionally, they have gained significant attention due to their favourable biochemical properties [11], including high thermostability [23], deep tissue penetration [24], and low immunogenicity [11]. Furthermore, nanobodies can be economically produced in appreciable quantities by bacterial expression systems, either in the periplasm or via cell‐free methods, facilitating the accurate formation of disulphide bonds within the nanobody structure [25, 26], unlike larger antibodies, which typically require production in costly eukaryotic expression systems [27]. Because of these favourable properties, nanobodies and their derivatives are increasingly utilised in numerous biochemical applications, where they can readily substitute conventional antibodies [28]. They are also being applied as novel tools to address research questions where conventional antibodies have failed, such as stabilising protein conformational states and dynamics [29, 30, 31, 32, 33, 34], as well as controlling allosteric modulation of G‐protein‐coupled receptors [35, 36, 37]. Nanobodies have been also used as a carrier protein for the facile detection of peptides binding to the immune checkpoint protein programmed death 1 (PD‐1) [38]. Legobodies (complexes consisting of a nanobody, a Fab, and a fusion protein) [39], as well as megabodies (nanobodies grafted onto globular rigid bacterial proteins) [40] are utilised to increase particle mass for the structure determination of small proteins by single‐particle cryo‐electron microscopy [40]. A recently developed approach named Nanobody‐NanoBRET (NanoB^2^) employs nanobodies as fluorescent probes in conjunction with NanoLuciferase‐based bioluminescence resonance energy transfer (NanoBRET) [41] to study ligand binding to membrane proteins [42]. The widespread biochemical utility of nanobodies necessitates the creation of new nanobodies capable of binding to a diverse array of molecular targets. Nanobodies can be conventionally obtained by immunising an animal, typically a camelid such as a llama or a camel, with the target antigen [43]. However, with the recent advances in directed evolution, libraries of nanobodies can be generated entirely synthetically within a matter of weeks [15]. These libraries typically vary in the length of the main CDR‐H3, resulting in three unique interaction surfaces: a concave, a protruding loop, and a convex‐shaped paratope [15]. They can be used to select binders against target proteins, including membrane proteins and rare conformational states [32].

Apart from their clear usefulness in basic biochemical research, nanobodies are increasingly employed as diagnostic tools [44, 45], molecular imaging probes [45], and therapeutic agents [44, 45, 46, 47]. They are currently under clinical investigation for a diverse range of human diseases [3], including conditions such as breast cancer [48], brain tumours [24], lung diseases [49], and infectious diseases [50]. Nanobodies can also target various tumours [9, 51] and are used in the diagnosis and treatment of prostate cancer [7]. Since 2019 and particularly with the COVID‐19 pandemic, several studies, including computational protein design, have emerged investigating the potential of nanobodies as antiviral agents [9, 51]. Nanobodies have been engineered to specifically target the receptor‐binding domain of the SARS‐CoV‐2 spike protein [52, 53, 54, 55, 56, 57, 58, 59], including that of the Omicron variants [14], to block its interaction with the human angiotensin‐converting enzyme 2. By binding to the spike protein, nanobodies interfere with the virus's ability to infect human cells and potentially neutralise its infectivity.

Despite the promising potential of nanobodies as alternatives to conventional antibodies, they do suffer from a few limitations. These include changes in their binding properties when labelled with imaging agents, high uptake of radiolabelled nanobodies in kidneys and liver, which complicates lesion detection and causes organ exposure, and rapid renal excretion of nanobodies, reducing efficacy in targeting disease sites [60]. The current availability of experimental nanobody–antigen structures is also quite limited. While experimental structural data remain invaluable, computational modelling and design offer a complementary approach to understanding nanobody–antigen interactions, especially when experimental structures are lacking. These methods accelerate the discovery, optimisation, and redesign of nanobodies with high specificity and affinity for a wide range of antigens. Computational and artificial intelligence‐based methods for antibody modelling [61], development [62, 63] and for protein design for COVID‐19 research and emerging therapeutics [9, 64, 65] have already been reviewed. For nanobodies, structural characteristics enabling precise and robust target binding have also been reviewed highlighting emerging technologies for identification, structural analysis, and humanisation [3]. Similarly, the different strategies that allow for rapid identification of target‐specific nanobodies and the engineering technologies that broaden their application [66] have been recently reviewed elsewhere. Here, we review computational modelling and design strategies to help leverage computational approaches in the development of next‐generation nanobody‐based therapeutics and biotechnological solutions. Specifically, we will highlight recent developments in computational approaches for nanobody‐antigen structural predictions, interactions, binding affinities, and nanobody design.

## Nanobody databases

Nanobody databases play an important role in providing resources and information for research, facilitating nanobody development, enabling target discovery and validation, and supporting comparative analysis. Most of the available databases primarily contain data for antibodies, with some also including information on nanobodies [62, 67]. In recent years, there has been a significant increase in the gathering of nanobody‐related data driven by advancements in both research and practical applications. This abundance of information has consequently fostered the expansion of databases specifically designed for nanobodies. The Integrated Database of Nanobodies for Immunoinformatics (INDI) contains more than 11 million nanobody sequences [68]. Its search tool finds the closest matching variable sequence in the INDI database, while the CDR‐H3 search tool helps locate nanobodies with similar CDR‐H3 regions as the query sequence. The Structural Antibody Database SAbDab‐nano is an explicit nanobody‐tracking sub‐database of SAbDab [69] that contains 1454 nanobody structures as of March 2024 and is updated weekly [70]. This is in addition to a non‐redundant dataset of 123 nanobody–antigen crystal structures, including their amino acid sequences and annotated CDR regions. Furthermore, interaction properties, including the number of intermolecular interactions, experimental binding affinities and changes in the solvent accessibility upon complex dissociation, are listed [71]. To address the challenges posed by heterogeneity, inconsistency, and the lack of interoperability among data across diverse databases, a novel database termed Nanobody Library and Archive System (NanoLAS) has been developed [72]. NanoLAS integrates and standardises nanobody data sourced from multiple databases, offering a user‐friendly, efficient, and interactive platform for data querying and analysis. In complement to existing structure and sequence databases, NbThermo serves as a pioneering database, compiling melting temperatures (*T*_m_) data for hundreds of nanobodies [73]. Its pivotal role extends to the development of algorithms for accurate *T*_m_ prediction in nanobody engineering and understanding the complex structural basis of nanobody thermostability. While there appear to be no apparent differences in the sequence pattern of the frameworks of nanobodies with lower and higher melting temperatures, it is evident that the highly variable loops play a crucial role in defining thermostability [74].

## Computational approaches for nanobody modelling

### Structural predictions of nanobody–antigen complexes

The prediction of nanobody structures continues to pose a challenge, in particular the accurate prediction of the CDR loop conformations [75]. Specifically, CDR‐H3, the most variable loop in length and amino acid composition, is the most difficult to predict [12, 21]. Several machine‐learning methods have been developed to facilitate nanobody structure prediction and design [63]. AlphaFold version 2.2 was used to model nanobody structures and was found to correctly predict the CDR‐H3 loop conformations when compared to experimental structures [18]. The recently developed tool ImmuneBuilder, predicts CDR‐H3 loops with an average RMSD of 2.9 Å, representing a 0.5 Å improvement over AlphaFold 2 [76]. NanoNet, trained specifically for nanobody structure prediction, offers rapid (a few milliseconds per nanobody) and efficient structural predictions allowing high‐throughput structure modelling [77]. To assess the global structural flexibility and local conformations of a nanobody, MD simulations were carried out on both an experimentally determined structure and a model predicted by NanoNet. Notably, CDR‐H1 and significant portions of CDR‐H3 converged on distinct conformations [78]. This underscores the complexity of nanobody modelling, emphasising that a static experimentally derived ‘snapshot structure’ may not comprehensively capture all the varied conformations nanobodies adopt. IgFold is a rapid and precise deep‐learning method designed to predict antibody and nanobody structures based on sequence information [79]. The accuracy of IgFold predictions aligns with recent AlphaFold 2 models, yet it operates at a significantly faster pace [75]. Evolvex is a recent *in silico* nanobody design pipeline that uses the empirical force field FoldX to design the CDR regions. Using this approach, high affinity, specific and stable nanobodies that target predefined epitopes on protein targets were designed [80]. AbNatiV, a newly developed deep learning tool, assesses the nativeness of antibodies and nanobodies, predicts immunogenicity likelihood and provides a residue‐level profile to guide the engineering of antibodies and nanobodies that closely resemble those derived from the immune system [81].

Another challenge is the prediction of antibody– and nanobody–antigen complex structures [82, 83] and binding affinities [83]. While predictive docking of nanobody–antigen complexes and the identification of interaction surfaces still remains challenging [83], a study showed that using the empirical Dreiding force field [84] to calculate the interaction energies in nanobody–antigen complexes is effective. This approach was particularly useful for reproducing the experimental binding poses predicted by the docking software zdock [85]. The data‐driven docking webserver Haddock [86], has also been widely used for predicting antigens binding to their nanobodies [87, 88, 89, 90, 91]. It demonstrates superior performance and produces models of higher accuracy compared to other docking methods [92]. HDOCK [93] was found to be the most suitable program for docking of novel nanobodies to the receptor‐binding domain of SARS‐CoV‐2 with high accuracy [94]. While RosettaAntibody [95, 96] is also being used to predict nanobody–antigen complexes [97, 98, 99], there is ongoing research aimed at improving its accuracy and speed of CDR‐H3 loop modelling [96]. PatchDock, which employs an algorithm capable of automatically detecting the CDRs of the antibody and restricts the search to these specific regions [100], has been integrated into a computational dock‐and‐design workflow [101]. In this workflow, the docking poses generated by PatchDock closely resembled the native ones [101]. A benchmarking study revealed that AlphaFold version 2.3.0 improved the success rate of near‐native nanobody–antigen modelling to 27%, compared to the 14% success rate for antibody–antigen complexes. Although the 27% success rate may still be considered low, it represents a notable improvement over the previous AlphaFold version 2.2 [102]. The better performance of modelling nanobody–antigen complexes versus antibody–antigen complexes was attributed to the lower number of CDR loops and, therefore, a smaller search space. Recently, a method involving the utilisation of AlphaFold 2 with aggressive sampling, known as AFsample, emerged as the best approach for multimer prediction in CASP15 [103]. It was able to improve the quality of the generated models (DockQ score = 0.55) by employing extensive sampling with a notable improvement compared to AlphaFold‐Multimer v2 (DockQ score = 0.41) [104]. During the final review of this review article, AlphaFold 3 was released [105]. It showcases notable advances in antibody–antigen prediction accuracy compared to AlphaFold‐Multimer v2.3.

#### Exploring nanobody and nanobody–antigen interactions using molecular dynamics simulations

Molecular dynamics (MD) simulations are valuable for investigating nanobody–antigen interactions and dynamics [106, 107, 108]. These simulations offer unique insights into identifying changes in the flexibility of CDR loops upon nanobody–antigen binding [101], and elucidating the molecular mechanisms underlying nanobody–antigen interactions, providing perspectives that complement experimental observations. Integrating computational and experimental approaches enhances our understanding of these interactions, thereby facilitating the rational design of nanobodies [89].

**Fig. 2:**
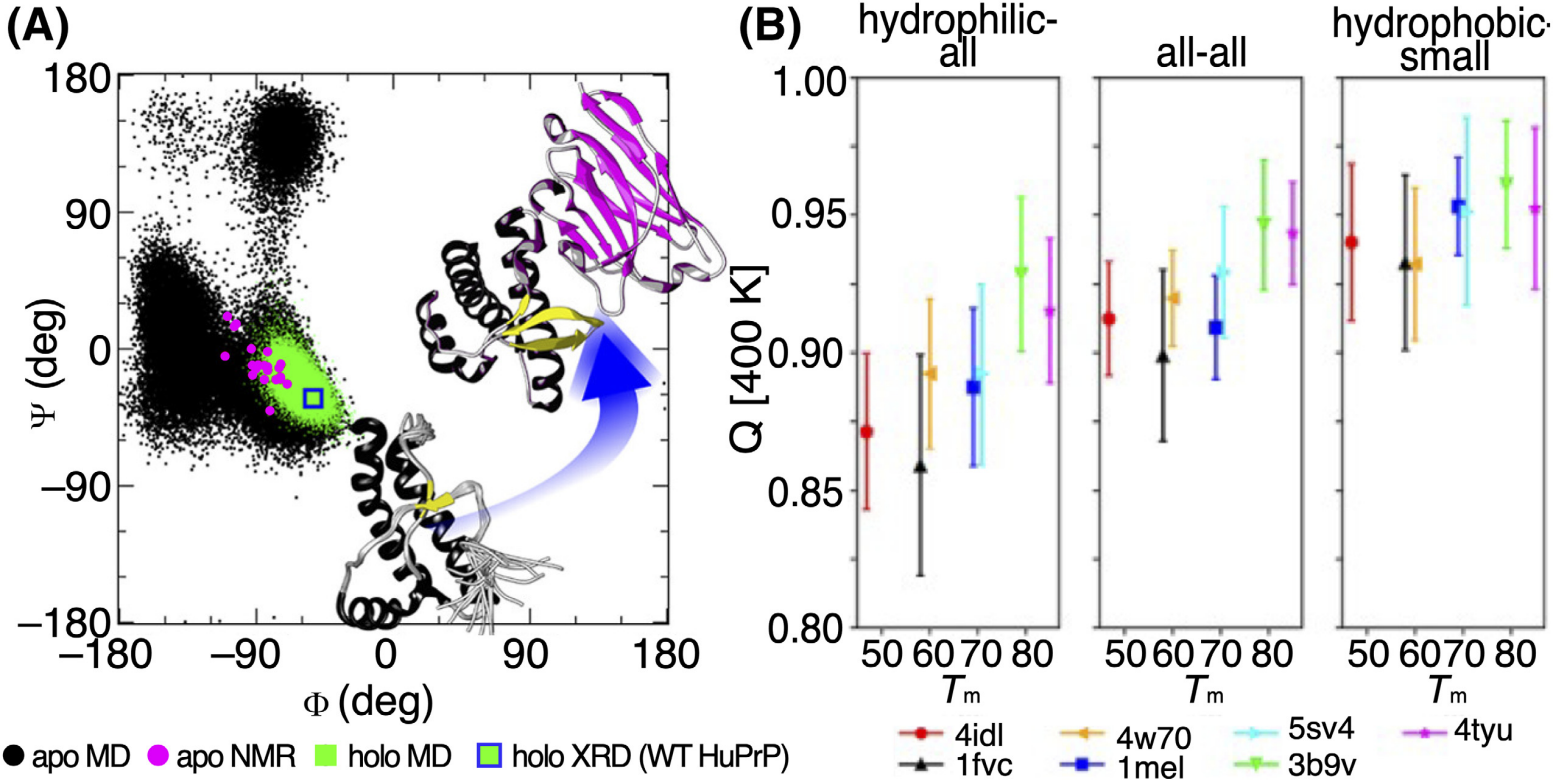
(A) Plot of Φ and Ψ dihedral angle distributions for a key residue Met166 involved in conformational changes of E219K HuPrP. In black dots, Φ and Ψ pairs from MD snapshots (*apo* MD) are reported; in pink dots, the ones extracted from the NMR structures (*apo* NMR); in green dots, the ones from simulated E219K HuPrP bound to the nanobody (*holo* MD); and in blue squares with green background, the ones extracted from the X‐ray crystallographic structure of WT HuPrP bound to the nanobody (*holo* XRD). Figure adapted from reference [112]. (B) Average native atomic contacts (*Q*) over the final 30 ns with standard deviation against the experimental *T*_m_ per group pair for the 400 K simulations. The hydrophilic‐all group is the average *Q* value between the hydrophilic residues (Asp, Glu, Gln, Asn, Arg, Lys, and His) versus all residues, the all–all group is the regular average *Q* value and the hydrophobic‐small group is the average *Q* value between the hydrophobic (Phe, Tyr, Trp, Leu, Val, Ile, Met, Cys, and Pro) versus the small (Gly, Ala, Ser, and Thr) residues. Figure adapted from reference [115].

MD simulations have been employed to explore the impact of linker length and flexibility on the architecture of bivalent nanobodies, comprised of two nanobodies connected by a linker [109]. The findings revealed that flexible linkers enhance the binding affinity of bivalent nanobodies irrespective of linker length, whereas rigid linkers require an ideal length for optimal performance [109]. They also facilitated the design of a novel nanobody probe for the detection of naturally occurring DNA G‐Quadruplex structures in human chromatin [110]. MD simulations have recently been used to understand the binding mechanism of a high‐affinity nanobody, designed to detect one of the earliest markers of human immunodeficiency virus infection, p24. The simulations have unveiled that binding takes place at a negatively charged region on p24, complemented by the positive surface of the nanobody's binding interface, which involves the CDR loops [111]. These simulations also underscored the significance of a salt bridge interaction, hydrogen bonding, and electrostatically complementary regions in facilitating the high‐affinity binding. Moreover, MD simulations have been employed in conjunction with existing NMR and X‐ray crystallography data on the human prion protein (HuPrP) and two of its disease‐linked mutants (E219K and V210I) [112]. These simulations elucidated the dynamic conformational landscapes of HuPrP and its mutants prior to binding to its associated nanobody. Previous studies have highlighted the nanobody's capability to inhibit prion aggregation *in vitro* by uniquely stabilising two disordered epitopes [112, 113]. During the simulations, experimentally determined binding‐competent conformations within the ensembles of pre‐existing conformational states were observed. This observation highlights the significance of a key residue Met166 in conformational changes and nanobody binding (Fig. 2A) [112]. Indirect of antigen binding, high‐temperature MD simulations of nanobodies revealed the importance of CDR‐H3 residues [114] and the interactions between CDR‐H3 and the framework residues [23, 114] for maintaining the VHH structural stability at high temperatures. Introducing mutations disrupting these interactions resulted in significant loss of affinity and thermal stability [23]. High‐temperature MD simulations (at 400 K) are believed to enhance the dynamics without perturbing the structures of the nanobodies [115]. The fraction of native atomic contacts (*Q*) displayed a good correlation with the experimentally determined melting temperatures (*T*_m_). *Q* values of hydrophilic residues exhibited an even better correlation, suggesting that nanobody stabilisation is correlated with favourable interactions of hydrophilic residues (Fig. 2B) [115].

#### Exploring conformational dynamics of nanobody–antigen complexes using enhanced sampling simulations

**Fig. 3:**
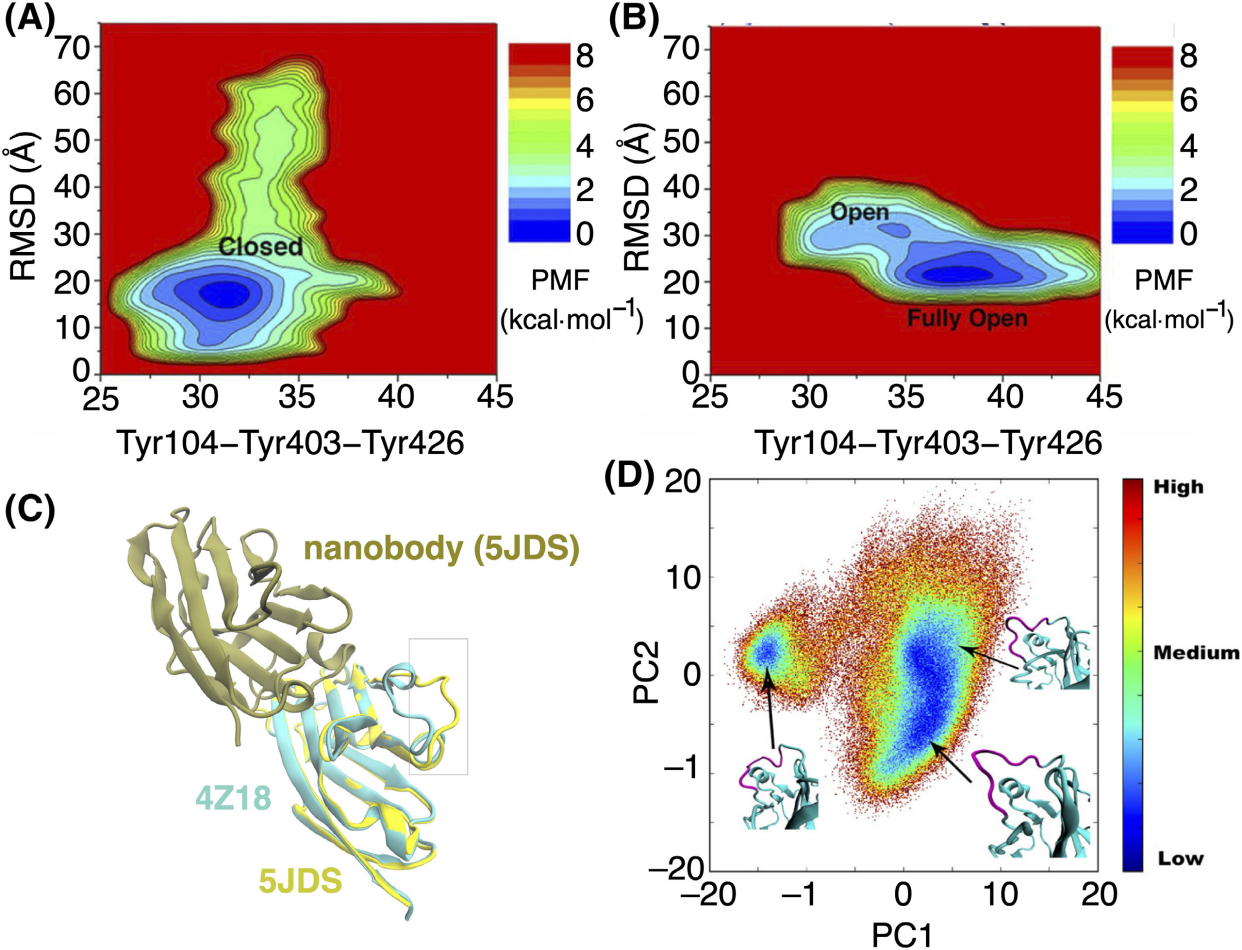
Two‐dimensional potential of mean force calculated using GaMD simulations between the Tyr104‐Tyr403‐Tyr426 triangle perimeter and RMSD of the nanobody relative to the X‐ray conformation (A) when the nanobody is bound showing the low‐energy conformational state as the closed and (B) when the nanobody is unbound showing two low‐energy conformational states as open and fully open. Figure adapted from reference [119]. (C) The superimposed structure of free PD‐L1 (PDB ID: 4Z18, cyan) over PD‐L1 cocrystallised with its nanobody (PDB ID: 5JDS, yellow). Figure adapted from reference [120]. (D) PC score plots of the PD‐L1 structures extracted from the GaMD simulations projected over the two largest principal components (PC1 and PC2). The figure gives representative structures of the low‐energy (high‐density) loop conformations. Figure adapted from reference [120].

Enhanced sampling simulations are used to explore the conformational space of nanobodies more efficiently and comprehensively than conventional MD simulations [116]. They aim to overcome the limitations of standard simulations, which may not adequately sample rare or high‐energy conformations. Gaussian accelerated MD (GaMD) is an enhanced sampling technique that works by applying a harmonic boost potential to smooth the energy surface, reduce the system energy barriers, and accelerate the structural dynamics by orders of magnitude [117]. GaMD simulations investigating nanobody binding to a G‐protein‐coupled receptor (GPCR) demonstrated that the orthosteric ligand‐binding pocket of the receptor underwent allosteric closure consistent with recent experimental findings (Fig. 3A) [118, 119]. In the absence of nanobody binding, the receptor's orthosteric pocket sampled both open and fully open conformations (Fig. 3B). These simulations provided valuable insights into the intricate mechanism of GPCR‐nanobody binding, showcasing the capability of GaMD in effectively modelling dynamic protein–protein interactions. Accelerated MD was also used to investigate the conformational dynamics of the binding domain of the immune checkpoint PD‐L1 [120]. The maximum structural displacements observed in both PD‐L1 crystal structures and MD trajectories were mainly by a specific loop, particularly when PD‐L1 is bound to its nanobody (Fig. 3C). Principal component (PC) score plots identified three regions with high density, and all of them represent open loop conformations (Fig. 3D). This highlights the potential benefits for targeting the region close to this flexible loop and could be a good target for allosteric small molecule ligands [120]. While GaMD improves sampling efficiency and reduces computational costs compared to conventional MD simulations, it encounters challenges related to insufficient sampling [121]. This hinders the calculation of converged free energy profiles required for studying large, complex systems or events spanning hundreds of milliseconds. To address this issue and maintain accurate free energy calculations, an integration of GaMD with replica exchange algorithms is proposed [121].

### Estimating the binding affinities of nanobody–antigen complexes

Estimating the binding affinities of nanobody–antigen complexes is a crucial aspect of understanding their interactions, optimising their therapeutic or diagnostic potential and identifying mutations that cause certain diseases. While experimental techniques are typically used as the gold standard to quantitatively assess binding affinity, computational methods can provide valuable insights into binding interactions which can facilitate the discovery of new or improved affinity binders, especially in cases where experimental data is limited or expensive to obtain. For instance, computational tools could be used to improve nanobody–antigen binding affinity by affinity maturation [101, 122], CDR‐swapping mutagenesis [123], or the design of multivalent nanobodies [2, 55, 58, 59, 109]. Alchemical binding free energy perturbation calculations were used to estimate the free energy changes of antigen binding caused by nanobody residue mutations [124, 125]. Binding free energies between the nanobody–antigen complexes could also be calculated following MD simulations using the Molecular Mechanics/Poisson Boltzmann Surface Area (MM/PBSA) [101, 112] or Molecular Mechanics/Generalised Born Surface Area (MM/GBSA) [110] methods or docking scoring functions [126]. Experimental measurements reflect average values of numerous binding events, while MD enables construction of an equilibrium ensemble for accurate assessment of nanobody–antigen complexes. Evaluating binding affinity based on this ensemble rather than a single configuration, especially for complexes lacking structural information, minimises potential errors [126]. A recently developed unsupervised binding energy prediction tool DSMBind outperformed most of the unsupervised approaches and matched the performance of supervised models despite not using any binding affinity labels during training [127]. Its design capability was showcased through PD‐L1 nanobody design task. Here, all three CDRs were randomised and the best CDR sequences were selected based on DSMBind score. The designed nanobodies were then tested experimentally and lead to the successful discovery of a novel PD‐L1 specific binder [127]. While computational methods for predicting nanobody– and antibody–antigen affinity have shown considerable advancements, there remains ongoing development to enhance accuracy further. This field is evolving rapidly, with increasing integration of machine‐learning algorithms [128, 129], suggesting a promising trajectory towards more precise and reliable affinity prediction in the future.

## Computational approaches for nanobody design

### Structure‐ and fragment‐based design of nanobodies

**Fig. 4:**
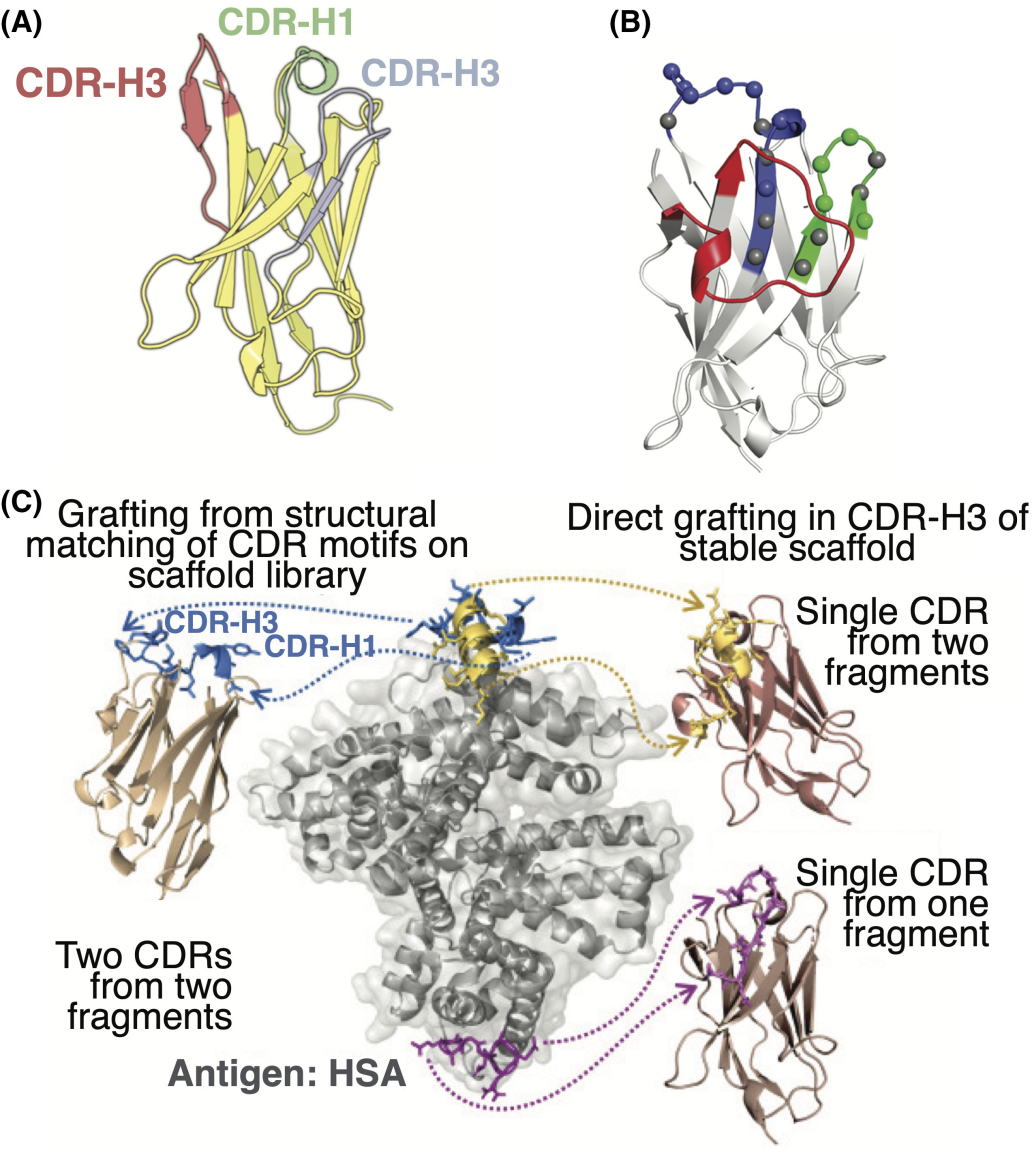
(A) X‐ray crystal structure of the WIW nanobody inhibitor showing its CDRs. CDR‐H1 in green, CDR‐H2 in blue, and CDR‐H3 in red (PDB ID: 8FQ7). Figure adapted from reference [130]. (B) AbBCII10 structure (PDB ID: 3DWT). CDR‐H1, CDR‐H2 and CDR‐H3 are coloured in blue, green, and red, respectively. Coloured spheres in CDR‐H1 and CDR‐H2 represent the randomised positions, while grey spheres represent CDR positions that were kept fixed. Figure adapted from reference [132]. (C) Grafting of designed CDR motifs onto antibody scaffolds. The structure of human serum albumin (HSA) is shown in grey, and the designed CDR motifs selected for experimental validation are shown in blue, yellow, and purple docked onto their respective epitopes. Two fragments (blue) are grafted into separate CDRs (CDR‐H1 and CDR‐H3) of a nanobody scaffold. The yellow and purple motifs are instead grafted into the CDR‐H3 of a scaffold resilient to CDR‐H3 substitutions. Figure adapted from reference [134].

Structure‐based design of nanobodies involves rational engineering and modification to enhance binding affinity, improve stability, reduce immunogenicity, fine‐tune biophysical properties, or tailor for specific applications. As an example, sequences of fibril‐capping amyloid inhibitors (VDW, W3 and WIW) were grafted onto a previously reported nanobody CDR‐H3 scaffold (PDB ID: 6HEQ) to halt tau aggregation linked to Alzheimer disease (Fig. 4A) [130]. Additionally, a bispecific nanobody combining a blood–brain barrier targeting nanobody (IR5, a nanobody that targets type 1 insulin‐like growth factor receptor) [131] with the WIW tau capping nanobody inhibitor joined by a flexible linker (Gly_4_Ser)_3_ was designed [130]. This nanobody demonstrated improved blood–brain barrier penetration suggesting a promising avenue for inhibiting prion‐like seeding of tau in neurodegenerative disorders. Design of a synthetic nanobody library is characterised by two crucial elements: framework selection and CDR design. The VHH framework derived from the recombinant anti‐chicken lysozyme nanobody cAbBCII10 that possess high stability was selected, CDR‐H1 and CDR‐H2 maintained the fixed length of cAbBCII10, while CDR‐H3 featured a 14‐unit loop to establish a convex binding site topology (Fig. 4B) [132]. Guided by the crystal structure analysis of cAbBCII10, positions for randomisation were identified, adjusting codon usage to ensure stability (Fig. 4B). This included retaining specific amino acids in key locations to promote stability, emphasising polarity at solvent‐exposed positions, and excluding destabilising amino acids. This design strategy could be used on other stable frameworks with different CDR‐H1‐3 lengths [132]. To overcome the limitation of AlphaFold 2 in predicting antibody–antigen structures [102], very recently, a fine‐tuned RoseTTAFold2 and RFdiffusion network has been developed to *de novo* design nanobodies [133]. The designed complex display accurate prediction of the CDR loops and overall binding orientation. This protocol is expected to be the foundation for structure‐based nanobody– and antibody–antigen design in the future [133]. In addition to structure‐based design of nanobodies, fragment‐based approaches, which involve the identification and optimisation of smaller antigen‐binding fragments within the nanobody sequence, could be used. Recently, a fragment‐based approach that involved the combinatorial design of nanobody binding loops and their grafting onto nanobody scaffolds has been developed (Fig. 4C) [134]. Biophysical characterisation has demonstrated that all designs exhibit stability and effectively bind their intended target human serum albumin with affinities in the nanomolar range. This strategy would facilitate the generation of lead nanobodies binding to preselected epitopes.

### Computational affinity maturation of nanobodies

**Fig. 5:**
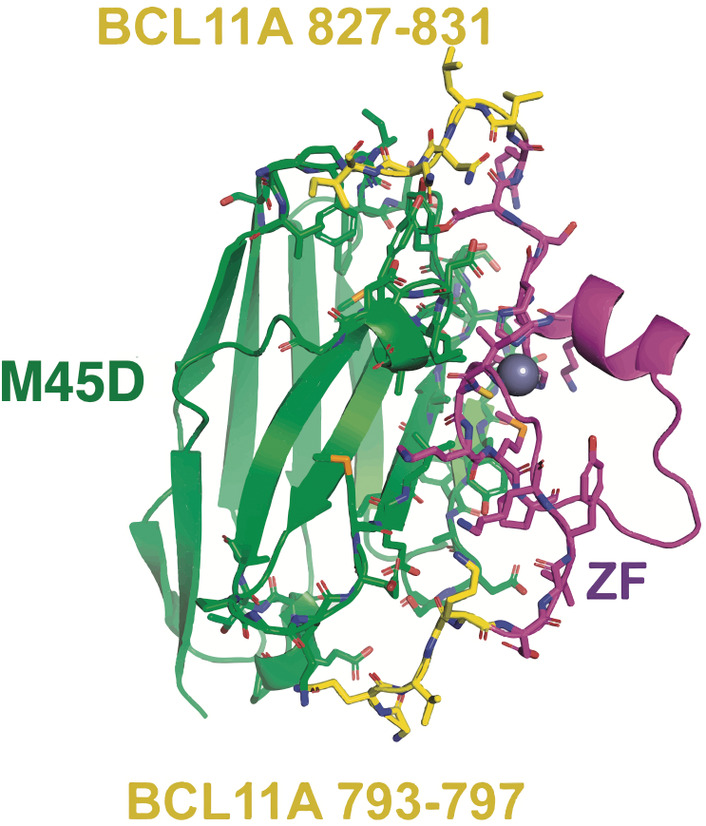
Structure of zinc finger (ZF) in complex with the M45D mutant nanobody and BCL11A. M45D is shown in green, ZF in magenta and BCL11A in yellow. Figure adapted from reference [141].

Computational affinity maturation of nanobodies refers to the process of using computational techniques to enhance the binding affinity of nanobodies by iteratively designing and optimising nanobody sequences or structures to improve their interactions with target antigens [122]. Computational methods enable the exploration of vast sequence and structural space to identify mutations [135, 136], modifications or non‐natural amino acid incorporations [107] that enhance nanobody binding affinity while maintaining specificity and stability [106]. A computational protocol based on MD simulations, molecular docking scores, FoldX stability prediction, CamSol and A3D solubility estimations resulted in accurate scoring methodologies for predicting experimental yields and identifying the structural modifications induced by mutations [137]. NanoBERT, a recently developed deep‐learning model, could be used to predict biologically feasible mutations in nanobodies based on their sequences [138]. The three‐dimensional structure of a nanobody targeting CD20, a phosphoprotein highly expressed on B‐cells in non‐Hodgkin lymphomas, was constructed using homology modelling, followed by molecular docking calculations to observe its interaction with CD20 [139]. After identifying the key residues, some mutations were introduced using the experimental design (Taguchi method) [140] aiming to improve the binding affinity of the nanobody to CD20. Following the mutations proposed by the experimental design, two optimised nanobody structures were developed, with one demonstrating notably enhanced binding affinity. MD simulations of this nanobody, whose sequence has been deposited in the INDI repository and patented (accession no. US20180079822), revealed that CDR‐H1 and CDR‐H3 are essential loops for recognising the antigen [139]. In a recent study, various experimental methods in conjunction with *in silico* protein design were used to develop specific nanobodies that recognise one of the C‐terminal zinc fingers of the transcription factor BCL11A, a pivotal regulator in the transition from fetal to adult‐type haemoglobin [141]. To enhance their affinity, loops were introduced before and after the zinc‐finger domain. The protein was then redesigned using rosetta software [142] by introducing mutations at the interaction interface (Fig. 5). Following the design phase, key metrics such as the rosetta score, RMSD, solvent‐accessible surface buried in contact, and change in binding energy, were assessed to rank the designs. Subsequently, it was discovered that a nanobody with the M45D mutation improved binding affinity experimentally [141]. In another study, the identification of mutations that should confer higher affinity to the original nanobodies was done, exploiting Venn‐intersection of multi‐algorithms screening (VIMAS). VIMAS is a method that combines the results obtained from three different platforms (mCSM‐AB, OSPREY, and FoldX), which use alternative algorithms to predict the effect of mutations on affinity [88]. In this strategy, each amino acid was sequentially mutated to each of 17 amino acids (excluding Cys and Pro), and the binding affinity change relative to the parent nanobody was calculated [90]. Binding free energies of the new nanobody–antigen complexes were then calculated using potential of mean forces from umbrella sampling simulations. This allowed the identification of a bispecific construct able to bind simultaneously the two clinically relevant antigens tumour necrosis alpha and interleukin 23 [90]. To accurately assess nanobody polyreactivity from protein sequences and predict the effects of amino acid mutations on polyreactivity, a machine‐learning model trained on a diverse naïve synthetic nanobody library has been developed recently [143]. In summary, the interdisciplinary application of computational and experimental methods has significantly advanced the affinity and specificity of nanobodies for therapeutic targets, offering promising prospects for the development of improved therapies. Implementing deep learning approaches holds the potential to significantly reduce the overall timeline of the *in silico* maturation pipeline for antibodies and nanobodies [122, 144]. However, the current limitation lies in the availability of sufficient data [122]. Therefore, benchmarking efforts would prove invaluable in gathering the requisite data to ascertain the true state‐of‐the‐art and pinpoint areas demanding focused research efforts.

### Computational design of Quenchbodies

In 2011, Ueda and co‐workers introduced an innovative biosensor known as Quenchbody (Q‐body). A Q‐body is a type of immunosensor designed for non‐competitive homogeneous assays targeting a range of antigens, including small molecules [145]. The pivotal aspect of this technology involves labelling the antibody fragment with a fluorescent dye, which is quenched by intrinsic exposed tryptophan residues in the antibody fragment. When these antibody fragments bind to antigens, the fluorescent dye molecule is sterically occluded and moves away from the quenching tryptophans, resulting in an increase in fluorescence intensity [145, 146]. Detecting antigens through fluorescence intensity changes is simple, easy to operate, and highly sensitive. The choice of the dye structure [147] the composition of the linker that attaches the dye to the antibody fragment (length and flexibility) [147, 148, 149], and the position of key quenching tryptophans [150, 151] has been studied for maximising the fluorescence quenching and antigen‐dependent de‐quenching. To date, various formats of Q‐bodies have been developed targeting antigens of interest. Among these are the scFv‐based Q‐bodies that detect the antidepressant fluvoxamine [149] and the proinflammatory cytokine tumour necrosis factor alpha [152]. Additionally, there are Fab‐based Q‐bodies (also referred to as ultra Q‐bodies) that detect the highly addictive psychostimulant methamphetamine [153], herbal cannabis [154] and amyloid β oligomers to aid the diagnosis of Alzheimer's disease [155]. Finally, nanobody‐based mini Q‐bodies that detect the small hapten methotrexate [147], lysozyme [156] and recombinant human growth hormone and its isoforms [150] have been developed. Fab‐based Q‐bodies generally exhibit a more substantial increase in fluorescence upon binding to the antigen compared to the scFv‐based ones [157]. Nanobody‐based Q‐bodies hold several advantages over scFv‐ or Fab‐based quenchbodies. They have higher stability, increased tolerance to mutation, and are easier to produce [146]. Antibody Fab and nanobody fragments are also amenable to conversion to coiled Q‐bodies (CQ‐body) by the augmentation of a stable coiled‐coil peptide pair comprised of E4 and K4 incorporated by a linker [158]. Association of the K3 or K4 coil with a covalently attached fluorophore strategically places the fluorescent dye in an ideal position for quenching, facilitating the development of fluorescent biosensors by non‐covalent peptide labelling [159].

**Fig. 6:**
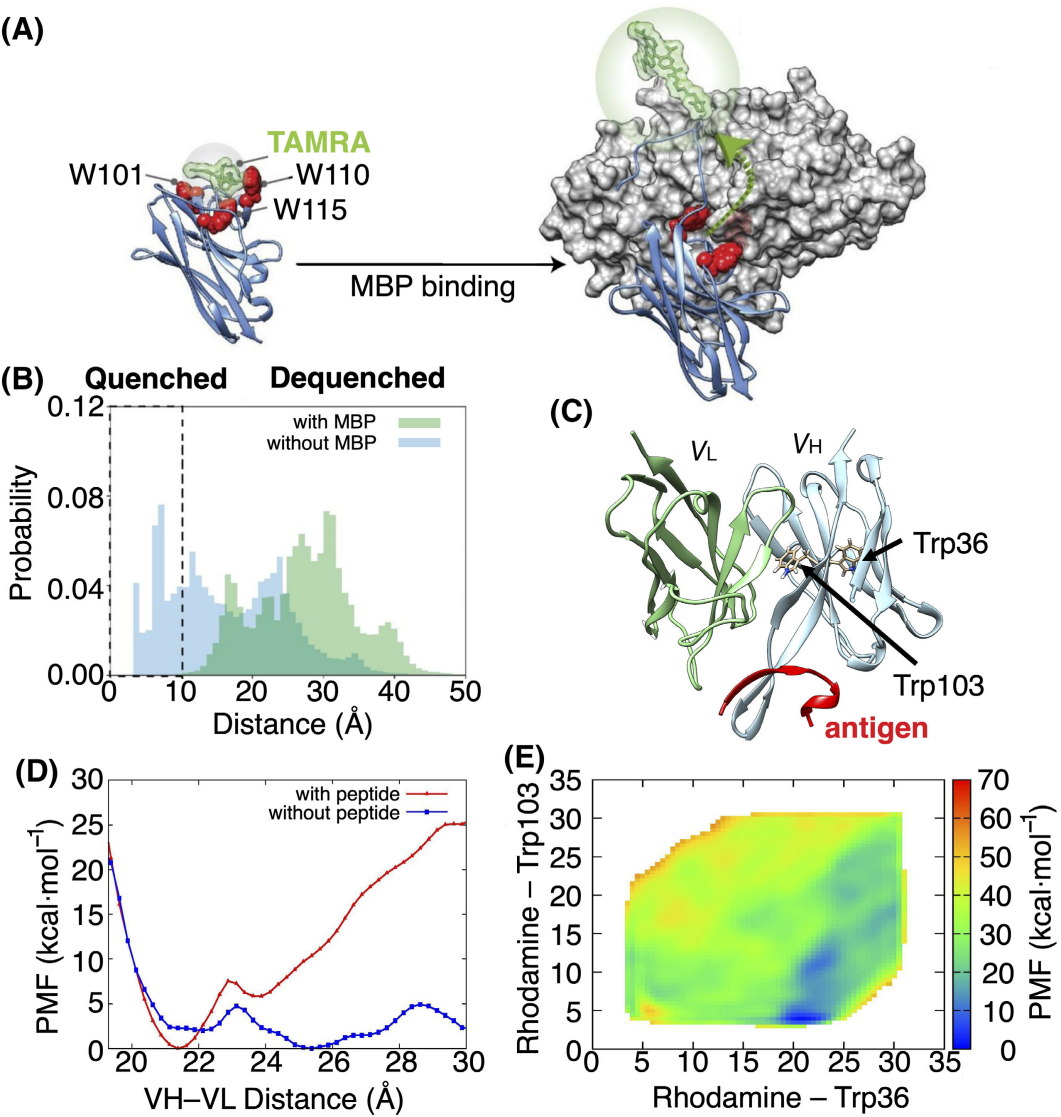
(A) Proposed mechanism for the MBP‐binding quenchbody (blue) modelled from PDB ID: 5M14, with covalently conjugated TAMRA at the N‐terminus (green) undergoing quenching due to interaction with the intrinsic CDR‐based tryptophans (red spheres). Upon binding to the MBP antigen (grey surface model), TAMRA is sterically occluded from tryptophans (W101, W110 and W115), which is associated with increased fluorescence intensity. Figure adapted from reference [151]. (B) Normalised distribution histograms illustrating the total TAMRA‐CDR‐tryptophan distances (W101, W110 and W115) derived from MD simulations in the absence (blue) or presence (green) of antigen for the MBP‐binding nanobody (PDB ID: 5M14). TAMRA is considered quenched by tryptophans at distances ≤ 10 Å (hatched zone). (C) Molecular structure of the VH and VL system. Trp36 and Trp103 in *V*_H_ (stick) and antigen molecule (red) are shown. (D) Potential of mean force as a function of the *V*_H_ − *V*_L_ distance. The red curve corresponds to the antibody with the antigen and the blue curve corresponds to the antibody without the antigen. (E) Potential of mean force as a function of the distance between the dye rhodamine and Trp36 and between rhodamine and Trp103. Figure adapted from reference [160].

The computational design of Q‐bodies has been explored minimally thus far. A promising avenue for future endeavours involves leveraging MD simulations for the design of high‐performance Q‐bodies. To our knowledge, the first *in silico*‐guided study to understand their quenching mechanism has been published only recently [151]. This study identified the key quenching tryptophans of nanobody‐based Q‐bodies of maltose‐binding protein (Qb‐MBP) and lysozyme (Qb‐Lys) (Fig. 6A). Through guidance provided by MD simulations (Fig. 6B), this study supports a working mechanism for nanobody‐based quenchbodies, whereby CDR‐based tryptophans that directly interface with antigens are the most important tryptophans for quenching of the dye [151]. Another computational study investigated the antigen‐dependent fluorescence response of a single‐chain scFv‐based quenchbody against Myc‐peptide antigen (Fig. 6C) [160]. The Myc‐peptide antigen is a peptide fragment derived from the c‐Myc protein, which is a transcription factor that plays a crucial role in regulating cell proliferation, growth, apoptosis, and differentiation. The free energy profile for the binding of the antigen to the variable heavy (VH) and light (VL) chains calculated from metadynamics MD simulations revealed that both chains bind in the presence of the antigen which seems to play an important role in binding [160] (Fig. 6D). Simulations also demonstrated that the fluorophore at the N‐terminus of VH interacts most stably with a key tryptophan (Trp103) (Fig. 6E). This provides computational support for the proposed experimental mechanism, where antigen presence buries tryptophan residues between VH and VL, eliminating fluorophore quenching [160].

## Conclusion

In conclusion, the field of nanobody research and design is witnessing remarkable advancements driven by computational approaches and experimental investigations. Nanobodies, characterised by their small size, high specificity, and stability, hold immense promise for various biomedical applications including diagnostics, therapeutics, and biosensing. The structural and functional insights into nanobodies, as elucidated by MD simulations and experimental studies, have paved the way for rational design strategies aimed at enhancing binding affinity, stability, and specificity. Through computational affinity maturation techniques, such as MD simulations and machine‐learning algorithms, researchers can iteratively optimise nanobody sequences to achieve desired properties and functionalities. Furthermore, the development of novel computational tools and databases, including those for structural prediction, antigen docking, and binding affinity estimation, continues to expand the repertoire of resources available for nanobody design and analysis. The emergence of innovative biosensing technologies like Q‐bodies that can detect small molecules and proteins, underscores the versatility and potential of nanobodies in diverse applications beyond traditional antibody‐based assays. The latter is just one example for how the computational design and optimisation of nanobodies is being applied for the development of improved nanobody‐based research capabilities. In summary, the integration of computational approaches with experimental methodologies is revolutionising the field of nanobody research, enabling the rapid and precise design of nanobodies with tailored properties for specific applications. As computational techniques continue to evolve and improve, they will undoubtedly play a pivotal role in accelerating the development and deployment of nanobodies across a wide range of biomedical and biotechnological domains, ultimately contributing to advancements in healthcare and diagnostics.

## Conflict of interest

The authors declare no conflict of interest.

## Author contributions

Conceptualisation: NSES, HY; funding acquisition: LMS, HY; visualisation: NSES, JHC, HY; writing‐first draft and reviewing literature: NSES; writing‐original draft: NSES, JHC, LMS, HY.
